# Genistein inhibits the proliferation and differentiation of MCF-7 and 3T3-L1 cells via the regulation of ERα expression and induction of apoptosis

**DOI:** 10.3892/etm.2014.1771

**Published:** 2014-06-10

**Authors:** EUN JEONG CHOI, JAE YEON JUNG, GUN-HEE KIM

**Affiliations:** Plant Resources Research Institute, Duksung Women’s University, Seoul 132-714, Republic of Korea

**Keywords:** genistein, 3T3-L1 cells, MCF-7 cells, proliferation, differentiation, estrogen receptor-α

## Abstract

The present study investigated the effect of the phytochemical genistein on the proliferation and differentiation of MCF-7 and 3T3-L1 cells via the regulation of estrogen receptor-α (ERα) expression and the induction of apoptosis. When MCF-7 human breast cancer cells were treated with 50, 100, 150 and 200 μM genistein for 24, 48 or 72 h, cell growth was significantly decreased in a concentration-dependent manner. Notably, the patterns of ERα expression and proliferation in MCF-7 cells treated with genistein were similar. Furthermore, ERα expression in differentiating 3T3-L1 cells was significantly inhibited by 48 h treatment with 50 μM genistein, which was selected based on the results of cytotoxicity assays on 3T3-L1 preadipocytes [lactate dehydrogenase (LDH) and 3-(4,5-dimethylthiazol-2-yl)-2,5-diphenyltetrazolium bromide (MTT) viability assays]. Under the same conditions, genistein-induced apoptotic features were observed in MCF-7 and differentiating 3T3-L1 cells. This observation is supported by the finding that B-cell lymphoma 2 (Bcl-2) expression was reduced while that of Bcl-2-associated X protein (Bax) was induced by genistein. The results of the present study suggest that an ERα-related pathway and the induction of apoptosis are involved in the proliferation of MCF-7 cells and the differentiation of 3T3-L1 cells.

## Introduction

Genistein (4′,5,7-trihydroxyisoflavone) is a major isoflavone present in plants, such as soybean, and therefore, in a variety of human foods (1–3). In 1987, it was discovered that genistein is a potent inhibitor of the tyrosine-specific protein kinase activity of the epidermal growth factor receptor (4). Since then, numerous researchers have studied the possible use of genistein as a cancer chemopreventive agent based on the key role of protein tyrosine kinase inhibitors in cancer cell growth and apoptosis (5,6). In support of these studies, several epidemiological reports have revealed significant correlations between genistein consumption and a reduced risk of breast cancer (7–9). Furthermore, a number of studies have demonstrated that genistein exhibits significant anticancer activity against breast tumors *in vitro* and *in vivo* (10,11).

Breast cancer belongs to a group of heterogeneous diseases with multiple clinical, molecular and histopathological forms, which makes achieving effective chemotherapy problematic (12). To develop breast cancer therapies, the targeting of estrogen receptor-α (ERα), which is expressed in ~70% of breast cancers and which makes it difficult to obtain a response to cancer drug treatment (13,14), requires consideration.

Therefore, the aim of the present study was to investigate the proliferative effects and induction of apoptosis by genistein via ERα-related pathways in MCF-7 human breast cancer cells and 3T3-L1 mouse preadipocytes.

## Materials and methods

### Reagents

All reagents and plasticware used for cell culture, including fetal bovine serum (FBS), media and antibiotics, were purchased from Invitrogen Life Technologies (Carlsbad, CA, USA) and Corning Incorporated Life Sciences (Corning, NY, USA). Insulin, dexamethasone, 3-isobutyl-1-methylxanthine (IBMX) and 3-(4,5-dimethylthiazol-2-yl)-2,5-diphenyltetrazolium bromide (MTT) were purchased from Sigma-Aldrich Co. (St. Louis, MO, USA). The protein analysis reagent and antibodies were purchased from Bio-Rad Laboratories, Inc. (Hercules, CA, USA) and Santa Cruz Biotechnology, Inc. (Santa Cruz, CA, USA), respectively. Genistein was purchased from LC Laboratories (Woburn, MA, USA) and dissolved in dimethyl sulfoxide (DMSO; final concentration of 0.1% in medium).

### Cell culture

The MCF-7 human breast cancer cells and 3T3-L1 mouse preadipocytes were purchased from the Korean Cell Line Bank (Seoul, South Korea) and American Type Culture Collection (Manassas, VA, USA), respectively, for use in the present study. The cells were maintained in Roswell Park Memorial Institute (RPMI)-1640 medium or Dulbecco’s modified Eagle’s medium (DMEM) supplemented with 10% FBS and antibiotics (50 U/ml penicillin and 50 μg/ml streptomycin) at 37°C in a humidified atmosphere containing 5% CO_2_. Two days subsequent to reaching confluence (designated as day 0), DMEM containing 10% FBS and differentiation inducers (10 μg/ml insulin, 0.5 μM dexamethasone and 0.5 mM IBMX) were added to the 3T3-L1 cells to induce differentiation.

### MCF-7 cell proliferation assay

MCF-7 cell proliferation was examined using MTT assays. Cells were plated at 2.5–5×10^5^ cells/well in a 96-well tissue culture plate and incubated for 24 h following which they were exposed to genistein solutions at concentrations of 50, 100, 150 and 200 μM. Following incubation for 24, 48 and 72 h, the plated cells were incubated with MTT (final concentration 0.5 mg/ml; Sigma-Aldrich) for 4 h at 37°C. The medium was discarded from the plates and 100 μl DMSO was added to each well. The plates were incubated for 5 min at room temperature whilst being shaken, so that the complete dissolution of formazan was achieved. The absorbance of MTT formazan was determined at 540 nm using an ultraviolet-visible (UV/VIS) spectrophotometric plate reader (EMax; Molecular Devices, LLC, Sunnyvale, CA, USA).

### Cytotoxicity assay using 3T3-L1 cells

Cellular toxicity was measured in 3T3-L1 preadipocytes using MTT and LDH assays with various concentrations of genistein (5–100 μM) for 24, 48 and 72 h. To measure lactate dehydrogenase (LDH) release, 100 μl/well supernatant medium was transferred to the corresponding well of an optically clear 96-well flat-bottom microtiter plate and analyzed using an LDH cytotoxicity detection kit (Takara Bio, Inc., Otsu, Japan).

### Apoptosis detection

Apoptotic morphological changes were identified by the 4′,6-diamidino-2-phenyl-indole (DAPI) staining of MCF-7 cells and differentiating 3T3-L1 cells, which had been treated with genistein at 50 μM for 48 h two days subsequent to reaching confluence. Each cell line was seeded on poly-L-lysine-coated slides and fixed with 4% methanol-free formaldehyde for 30 min. Mounting medium containing DAPI was dispersed over the entire slide. The mounted slides were stored at 4°C in the dark. Each slide was observed under an LSM700 laser scanning microscope equipped with Zen 2011 software (Carl Zeiss Microscopy GmbH, Jena, Germany).

### Immunoblotting

Following the exposure of MCF-7 and differentiating 3T3-L1 cells to genistein, each group of cells was subjected to lysis in radio-immunoprecipitation assay (RIPA) buffer [1% nonyl phenoxypolyethoxylethanol (NP)-40, 150 mM NaCl, 0.05% deoxycholic acid (DOC), 1% sodium dodecyl sulfate (SDS) and 50 mM tris(hydroxymethyl)aminomethane (Tris), pH 7.5] containing protease inhibitors for 1 h at 4°C. The supernatant was separated by centrifugation and the protein concentration was determined using a Bradford protein assay kit 2 (Bio-Rad). The proteins were then transferred to nitrocellulose membranes (0.45 μm). The membranes were blocked with 1% bovine serum albumin (BSA) for 1.5 h, washed twice with phosphate-buffered saline (PBS) containing 0.2% Tween-20, and incubated with the respective primary antibodies [cyclin D1, anti-ERα, -B-cell lymphoma 2 (-Bcl-2), -Bcl-2-associated X protein (-Bax) and -β-actin; Santa Cruz Biotechnology, Inc.] overnight at 4°C. The next day, the immunoreaction was continued using secondary rabbit anti-rabbit horseradish-peroxidase-conjugated antibodies following washing for 2 h at room temperature. Bands were detected with MicroChemi (DNR Bio-Imaging System, Ltd., Jerusalem, Israel) using WesternBright^™^ ECL solution (Advansta Inc., Menlo Park, CA, USA).

### Statistical analyses

All values are expressed as means ± standard deviations. Data were analyzed by an unpaired Student’s t-test or one-way analysis of variance followed by Dunnett’s multiple comparison test (Sigma Stat software; Jandel Scientific Software, San Rafael, CA, USA). For all comparisons, P<0.05 was considered to indicate a statistically significant difference.

## Results

### Antiproliferative activity of genistein toward MCF-7 cells

**Figure 1:**
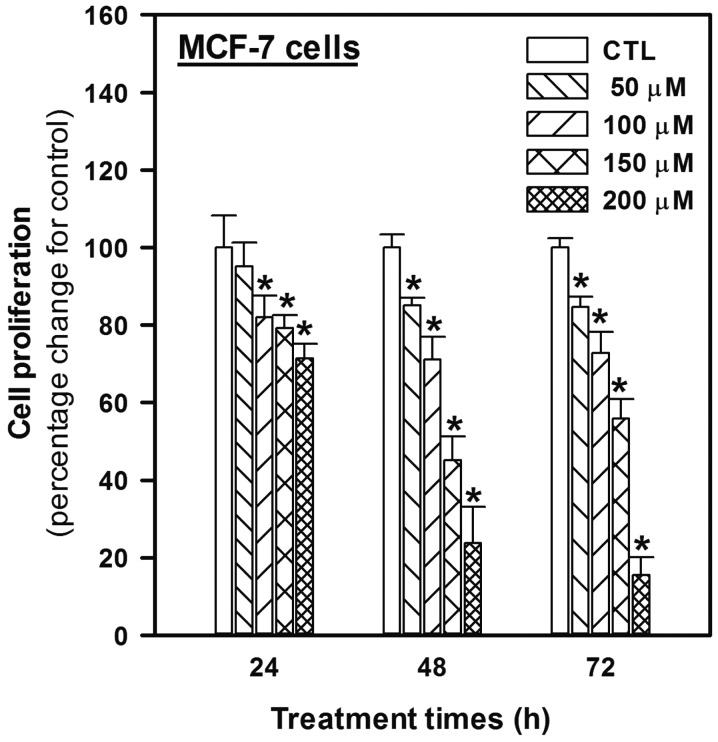
Antiproliferative effects of genistein on MCF-7 cells. MCF-7 cells were exposed to genistein at 50, 100, 150 and 200 μM for 24, 48 and 72 h. All data are reported as the percentage change, as compared with the control (CTL) group (vehicle only). Values are expressed as mean ± standard deviation. ^*^P<0.05, significantly different from the CTL group.

To investigate the possible anticancer effects of the phytochemical genistein on MCF-7 human breast cancer cells, the present study initially examined the antiproliferative effects of genistein on MCF-7 cells using various concentrations (50, 100, 150 and 200 μM) of genistein for 24, 48 and 72 h (Fig. 1). Genistein inhibited the growth of MCF-7 cells in a concentration-dependent manner and revealed significant antiproliferative activity under all treatment conditions, with the exception of 50 μM for 24 h. The antiproliferative activity of genistein after 48 and 72 h was stronger than that after 24 h, but there were no differences between 48 and 72 h; reductions of 13, 29, 55 and 77% at 48 h and 15, 27, 45 and 85% at 72 h for 50, 100, 150 and 200 μM genistein, were noted, as compared with the control levels after 48 and 72 h.

### Cytotoxicity of genistein toward 3T3-L1 preadipocytes

**Figure 2:**
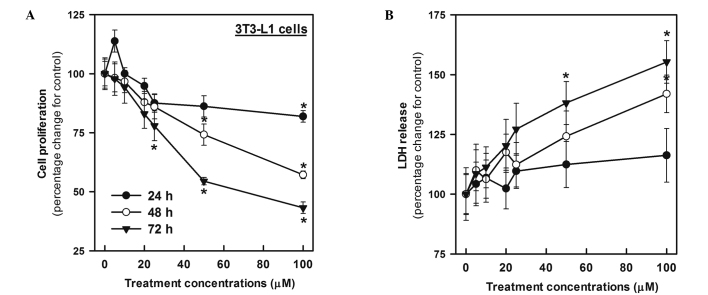
Cytotoxicity of genistein toward 3T3-L1 preadipocytes. (A) 3-(4,5-Dimethylthiazol-2-yl)-2,5-diphenyltetrazolium bromide (MTT) assay. (B) Lactate dehydrogenase (LDH) assay. 3T3-L1 preadipocytes were exposed to genistein at 5, 10, 20, 25, 50 and 100 μM for 24, 48 and 72 h. Values are expressed as means ± standard deviation. ^*^P<0.05, significantly different from the vehicle-only group.

To assess the cytotoxicity of genistein, cell growth and LDH release were measured in 3T3-L1 cells exposed to genistein at 5–100 μM for 24, 48 and 72 h. Genistein significantly decreased cell growth after 48 and 72 h in a concentration-dependent manner [the half maximal inhibitory concentration (IC_50_) for 48 and 72 h was 111.67 and 77.1 μM, respectively; Fig. 2]. Moreover, under the same conditions, exposure to genistein for 24 h caused LDH release to increase by 4–16%; however, the increase was not statistically significant. After 48 and 72 h, although the release of LDH increased in a concentration- and time-dependent manner, significant inhibition of cell growth was first observed in cells treated with 100 and 50 μM genistein for 48 and 72 h, respectively (Fig. 2).

### ERα, cyclin D1, and Bcl-2 expression in genistein-treated MCF-7 or differentiating 3T3-L1 cells

**Figure 3:**
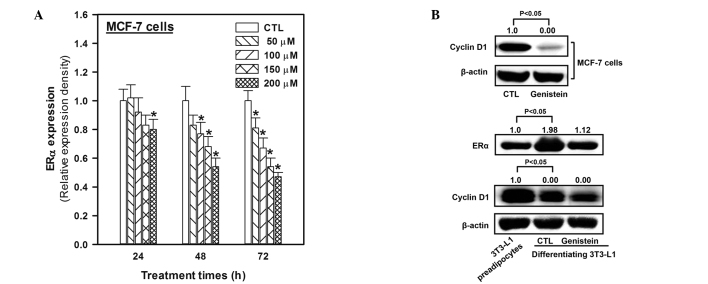
Modulation of estrogen receptor-α (ERα) and cyclin D1 by genistein in MCF-7 and differentiating 3T3-L1 cells. (A) For determination of ERα, MCF-7 cells were exposed to genistein at 50, 100, 150 and 200 μM for 24, 48 and 72 h. (B) In addition, 3T3-L1 and MCF-7 cells were exposed to 50 μM genistein for 48 h. Values are expressed as means ± standard deviations. CTL, control group (vehicle only); ^*^P<0.05, significantly different from the CTL group.

To elucidate the mechanism of the ERα-dependent antiproliferative activity of genistein, cells were exposed to 50, 100, 150 and 200 μM genistein for 24, 48 and 72 h. The results revealed that the patterns of ERα expression and proliferation were similar (Fig. 3). ERα expression was downregulated by genistein at all concentrations; furthermore, the effect of genistein was greater after 48 and 72 h. ERα expression was upregulated 1.98-fold in 3T3-L1 cells following inducer treatment for 48 h as compared with that in the negative control (3T3-L1 preadipocytes, Fig. 3), in order to initiate differentiation. Genistein treatment at a concentration of 50 μM for 48 h, which was selected as the effective (no significant cellular toxicity) concentration, restored ERα expression to almost the initial differentiating levels.

Cyclin D1 expression in MCF-7 and 3T3-L1 cells was decreased by treatment with genistein at 50 μM for 48 h (Fig. 3).

### Apoptosis inducing effect of genistein on MCF-7 and differentiating 3T3-L1 cells

**Figure 4:**
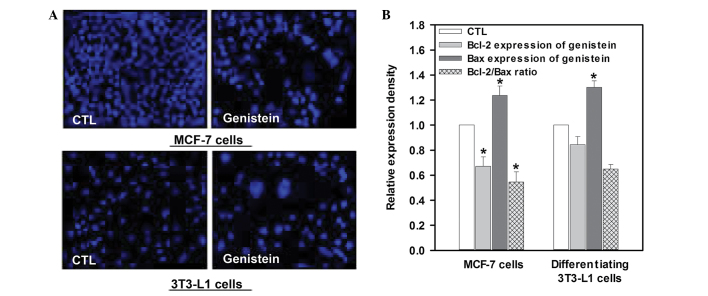
Induction of apoptosis by genistein. Apoptotic changes were detected by (A) 4′,6-diamidino-2-phenyl-indole (DAPI) staining and (B) measurement of the B-cell lymphoma 2 (Bcl-2)/Bcl-2-associated X protein (Bax) ratio. The standard deviations in all the control groups for MCF-7 and 3T3-L1 cells are <0.12. Values are expressed as means ± standard deviations. CTL, control group (vehicle only); ^*^P<0.05, significantly different from the CTL group.

As shown in Fig. 4, exposure of MCF-7 cells to genistein at a concentration of 50 μM for 48 h produced apoptotic morphological features, including cell shrinkage and dot-shaped nuclear fragments. Compared with the control cells, exposure to genistein resulted in an increase in apoptotic morphological features on both MCF-7 and differentiating 3T3-L1 cells. This result is supported by the fact that genistein significantly decreased the Bcl-2/Bax expression ratio by 45.5% and 35.2% in MCF-7 and differentiating 3T3-L1 cells, respectively, compared with their respective control levels (P<0.05, Fig. 4).

## Discussion

In the present study, the antiproliferative effects of genistein on MCF-7 cells at concentrations of 50, 100, 150 and 200 μM were investigated. Genistein is a phytoestrogen, a plant-derived phenolic compound that structurally mimics the hormone 17β-estradiol (15). Thus, the effects of genistein on estrogen receptors (ERs) are both agonistic and antagonistic (16,17), and may explain the biphasic effect of genistein on MCF-7 cell proliferation. A previous study (18) revealed that the antiproliferative effect of genistein on MCF-7 cells was biphasic (inhibitory at high concentrations and stimulatory at low concentrations). This result is consistent with that of other previous studies demonstrating that estrogen-like bioactive molecules may stimulate breast cancer cell growth (19–21). In the present study, following treatment for 24, 48 and 72 h, genistein significantly inhibited the proliferation of MCF-7 cells in a concentration-dependent manner. Notably, ERα expression was downregulated while proliferation was inhibited in MCF-7 cells exposed to genistein.

ERα is a member of the steroid receptor superfamily that regulates processes such as growth and differentiation in various target cells by affecting transcription. ERα also plays an important role in the development and progression of breast cancer. A novel strategy for breast cancer chemotherapy is the identification of ERα regulators among phytoestrogens (22,23).

Obesity is associated with an increased risk of developing cancer; in particular, obesity plays an important role in the pathogenesis of breast cancer since it causes altered adipokine levels, elevated circulating estrogen levels and insulin resistance (24). The differentiation of 3T3-L1 preadipocytes results in cells with the biochemical characteristics of adipocytes (for example, increased ERα expression). Upon the induction of differentiation in 3T3-L1 cells following inducer treatment for 48 h, ERα expression was significantly downregulated by genistein to an extent similar to that in 3T3-L1 preadipocytes. Based on these results, genistein may be associated with the proliferation of MCF-7 cells and differentiation of 3T3-L1 cells via ERα expression.

When genistein was applied to MCF-7 and 3T3-L1 cells, cyclin D1 expression was reduced. Cyclin D1 is a prominent target of estrogens in breast cancer cells and its induction is important for the progression of cells through the G1 phase of the cell cycle (25). The results of several studies suggest that cyclin D1 is overexpressed in breast cancer (26,27) and that it is associated with ER positivity in breast cancer (28–30).

Furthermore, the induction of apoptosis by genistein is supported by evidence demonstrating that the apoptotic cell population was increased in MCF-7 and 3T3-L1 cells. Apoptosis is essential for tissue development and homeostasis. The mechanism of apoptosis involves a balance between factors that induce and those that inhibit the process. Pro-apoptotic agents have been proposed as a novel strategy not only for cancer chemotherapy but also for the treatment of obesity (31). It has been reported (32,33) that the induction of apoptosis in adipocytes, which are otherwise resistant to apoptosis due to high levels of Akt/protein kinase B and Bcl-2, may be a method of reducing adipocyte numbers. In MCF-7 and 3T3-L1 cells, genistein treatment significantly reduced the Bcl-2/Bax ratio by decreasing Bcl-2 expression and increasing Bax expression. Bax genes and members of the Bcl-2 family, such as Bcl-2, are involved in the control of apoptotic pathways (34). The decreased expression of Bcl-2 and increased expression of Bax is associated with the response of cancer cell lines to anticancer compounds. In addition, a loss of Bcl-2 expression may promote the induction of apoptosis.

Based on the results of the current study, genistein inhibits the proliferation of MCF-7 and differentiation of 3T3-L1 cells via apoptosis induction and an ERα-related pathway. The effects of genistein observed in the present study make it potentially useful for further development as not only a chemotherapeutical agent for breast cancer but also a chemopreventive agent for obesity.
